# Properdin Is a Key Player in Lysis of Red Blood Cells and Complement Activation on Endothelial Cells in Hemolytic Anemias Caused by Complement Dysregulation

**DOI:** 10.3389/fimmu.2020.01460

**Published:** 2020-07-22

**Authors:** Jin Y. Chen, Neeti S. Galwankar, Heather N. Emch, Smrithi S. Menon, Claudio Cortes, Joshua M. Thurman, Samuel A. Merrill, Robert A. Brodsky, Viviana P. Ferreira

**Affiliations:** ^1^Department of Medical Microbiology and Immunology, University of Toledo College of Medicine and Life Sciences, Toledo, OH, United States; ^2^Department of Foundational Medical Studies, Oakland University William Beaumont School of Medicine, Rochester, MI, United States; ^3^Department of Medicine, University of Colorado Anschutz Medical Campus, Aurora, CO, United States; ^4^Section of Hematology/Oncology, Department of Medicine, West Virginia University School of Medicine, Morgantown, WV, United States; ^5^Division of Hematology, Department of Medicine, John Hopkins University School of Medicine, Baltimore, MD, United States

**Keywords:** complement, alternative pathway, properdin, monoclonal antibodies, aHUS, PNH, hemolysis, endothelial cell

## Abstract

The complement system alternative pathway (AP) can be activated excessively in inflammatory diseases, particularly when there is defective complement regulation. For instance, deficiency in complement regulators CD55 and CD59, leads to paroxysmal nocturnal hemoglobinuria (PNH), whereas Factor H mutations predispose to atypical hemolytic uremic syndrome (aHUS), both causing severe thrombohemolysis. Despite eculizumab being the treatment for these diseases, benefits vary considerably among patients. Understanding the molecular mechanisms involved in complement regulation is essential for developing new treatments. Properdin, the positive AP regulator, is essential for complement amplification by stabilizing enzymatic convertases. In this study, the role of properdin in red blood cell (RBC) lysis and endothelial cell opsonization in these AP-mediated diseases was addressed by developing *in vitro* assays using PNH patient RBCs and human primary endothelial cells, where the effects of inhibiting properdin, using novel monoclonal antibodies (MoAbs) that we generated and characterized, were compared to other complement inhibitors. In *in vitro* models of PNH, properdin inhibition prevented hemolysis of patient PNH type II and III RBCs more than inhibition of Factor B, C3, and C5 (>17-fold, or >81-fold, or >12-fold lower molar IC_90_ values, respectively). When tested in an *in vitro* aHUS hemolysis model, the anti-properdin MoAbs had 11-fold, and 86-fold lower molar IC_90_ values than inhibition of Factor B, or C3, respectively (*P* < 0.0001). When comparing target/inhibitor ratios in all hemolysis assays, inhibiting properdin was at least as efficient as the other complement inhibitors in most cases. In addition, using *in vitro* endothelial cell assays, the data indicate a critical novel role for properdin in promoting complement activation on human endothelial cells exposed to heme (a hemolysis by-product) and rH19-20 (to inhibit Factor H cell-surface protection), as occurs in aHUS. Inhibition of properdin or C3 in this system significantly reduced C3 fragment deposition by 75%. Altogether, the data indicate properdin is key in promoting RBC lysis and complement activation on human endothelial cells, contributing to the understanding of PNH and aHUS pathogenesis. Further studies to determine therapeutic values of inhibiting properdin in complement-mediated diseases, in particular those that are characterized by AP dysregulation, are warranted.

## Introduction

Complement is an essential component of host defense by being central to the innate and adaptive immune functions. It comprises three pathways, the classical, lectin, and alternative pathways that are each activated through different mechanisms and converge at the generation of C3b, which is then amplified by the alternative pathway (AP) [reviewed in (1)]. Complement activation leads to the formation of C3 fragments, which are important for opsonization and phagocytosis of microbes and dying cells and are also essential for adaptive immune responses, the release of chemotactic fragments (C3a, C5a) and the formation of membrane attack complex (MAC, C5b-9). Complement negative regulatory proteins are present on the host cell surfaces (CD46, CD55, CD59, CD35, CRIg, CSMD1), or in the fluid phase (Factor H, Factor H-like protein 1, Factor H-related proteins 1-5, clusterin, vitronectin, C1 inhibitor, and C4b binding protein) to control unnecessary complement activation [reviewed in (2)]. Although complement is essential for normal inflammatory processes, excess activation leads to or promotes heart, autoimmune and other diseases [reviewed in (3–5)], and even normal complement activity becomes detrimental in the context of defects in negative complement regulatory proteins [reviewed in (6, 7)].

Properdin is a highly positively charged glycoprotein that is the only known positive regulator of the complement system. The concentration of properdin in serum is 4–25 μg/ml and is present mainly as dimers (P_2_), trimers (P_3_), and tetramers (P_4_) of head-to-tail-associated 53 kDa monomers, in a 1:2:1 ratio [reviewed in (8)]. It stabilizes the C3 and C5 convertases, such that their activity increases 5-10-fold, thus leading to efficient amplification of C3b deposition on the cell surfaces [reviewed in (8)]. In addition to its role as a regulator of the AP, properdin can also act as an initiator of the AP by selectively binding to target surfaces and providing a platform for *de novo* convertase assembly (9). Examples of such cell surfaces include zymosan (10), late apoptotic (11) and necrotic cells (10), *Chlamydia pneumoniae* (12), and activated platelets (13). The role of properdin in diseases has been recently extensively reviewed in (14). Deficiency of properdin leads to susceptibility to bacterial infections, especially to severe fulminant meningococcal infections, which can be prevented by vaccination [reviewed in (15)]. Although properdin is essential for normal inflammatory processes, it also plays an important role in pathogenic processes such as thromboinflammation [reviewed in (16)], especially in the context of complement dysregulation. Examples using human systems include its role in enhancing platelet granulocyte aggregate formation in thrombin receptor-activated whole blood when Factor H regulation is disrupted (17, 18), and in promoting red blood cell (RBC) damage in paroxysmal nocturnal hemoglobinuria (PNH) (19), among others [reviewed in (14)].

PNH is an acquired defect of pluripotent hematopoietic stem cells in which mutations in the *PIG-A* (phosphatidylinositol glycan class A) gene result in a deficiency of glycosylphosphatidylinositol (GPI) anchor, thus preventing the presence of any of the proteins that require this anchor on the cell membrane, including CD55 and CD59, thus making derived RBCs highly sensitive to complement-mediated lysis [reviewed in (6)]. Somatic mutations in *PIG-A* can lead to cells lacking GPI linked proteins (type III cells), and cells with intermediate levels of GPI linked proteins (type II cells) (20). Instead of being eliminated by complement immediately, PNH type III RBCs circulate in the blood for variable amounts of time [6–120 days; (21, 22)], and the lifespan of a particular PNH cell will depend on the stochastic nature of C3 deposition (23). In addition, Factor H contributes to preventing the immediate lysis of PNH RBCs by contributing to protecting the RBCs surface from complement-mediated lysis (24). Atypical hemolytic uremic syndrome (aHUS) is another prototype disease of excessive complement activation during complement dysregulation. One of the major causes of aHUS is mutations in the C terminal domains 19–20 of Factor H, which lead to impaired binding of Factor H to cell surfaces and result in ineffective control of complement activation [reviewed in (7)]. Hemolysis and thrombosis are the two primary causes of morbidity in PNH [reviewed in (6)] and aHUS [reviewed in (7)].

Inhibition of complement components is being evaluated for clinical applications for PNH and aHUS. This includes blockade of complement proteins involved in activation and amplification (C3, Factor B, or Factor D) or complement terminal pathway component C5 for PNH [reviewed in (25–27)], and blockade of initiation molecule MASP2 or terminal pathway components (C5 or C5aR1) for aHUS [reviewed in (25, 27)]. Eculizumab is the complement-specific inhibitor that is approved for PNH [reviewed in (26)] and aHUS (28). An improved version of eculizumab, ravulizumab, was recently approved for PNH treatment (29). Both agents prevent cleavage of C5, thus effectively blocking the generation of the pro-inflammatory molecule C5a and formation of the terminal complement MAC (30). Eculizumab has significant benefits on PNH (31) and aHUS (28) patients. However, it is one of the most expensive drugs on the market [reviewed in (32)]. Moreover, suboptimal responses by eculizumab in PNH (33) and aHUS (34) patients have been reported. Eculizumab cannot inhibit C3b opsonization and this high density of surface C3b molecules will compete with the ability of the C5 inhibitor to bind to C5 (35), leading to lack of inhibition of MAC formation, residual intravascular hemolysis and incomplete clinical responses, leaving patients transfusion-dependent (33). The unimpaired C3b opsonization and subsequent formation of C3dg due to the intrinsic limitation of C5 inhibition lead to accelerated removal of RBCs by extravascular hemolysis (36–38). In addition, PNH patients with C5 mutations have a poor response to eculizumab (39), and results of clinical trials for rheumatoid arthritis [reviewed in (32)] and cardiovascular disease (40) were disappointing. Hence, there is a need to further define molecular mechanisms involved in complement regulation in human disease processes in order to find alternatives to the existing treatment options.

The importance of properdin in disease has also been demonstrated using properdin-deficient mice disease models of asthma (41), arthritis (42–44), abdominal aortic aneurysm formation (45), *Streptococcus pneumoniae*-induced septicemia (46), renal ischemia reperfusion injury (47), 5-fluorouracil-induced small intestinal mucositis (48), zymosan-induced non-septic shock (49) and aHUS (50), where the properdin-deficient mice were more protected from the disease vs. wild type animals [reviewed in (14)]. While lack of properdin often protects host from exacerbated inflammation, lack of properdin in mice can be deleterious in other disease models such as colitis (51, 52), *Listeria monocytogenes*-induced septicemia (46), polymicrobial septic peritonitis (53), LPS-induced non-septic shock (49), and C3 glomerulopathy where Factor H is also absent in the mice (54, 55). Thus, the exact role of properdin in the pathogenesis of diseases needs to be assessed in a disease-specific and species-specific manner. Hence, we developed and characterized monoclonal antibodies (MoAbs) against human properdin and studied the functions of properdin in PNH- and aHUS-like *in vitro* human disease model assays developed in our laboratory. By stimulating endothelial cells with hemolysis-derived heme and rH19-20, a competitive inhibitor of Factor H cell surface protection (56), or with aHUS-related mutants (57), in the presence of human serum, we established an *in vitro* endothelial cell model to mimic aHUS conditions. We analyzed the effects of inhibiting properdin and blockade of other complement components, including Factor B, C3 and C5, elucidating crucial and novel roles for properdin in amplifying complement activation and mediating damage of human RBCs and endothelial cells in PNH and aHUS.

## Materials and Methods

### Buffers

Gelatin veronal buffer (GVB=) (5 mM veronal, 145 mM NaCl, 0.004% NaN_3_, 0.1% gelatin, pH 7.3), GVBE [GVB= with 10 mM EDTA (ethylene diamine tetraacetic acid)], MgEGTA [0.1 M MgCl_2_ and 0.1 M EGTA (ethylene glycol tetraacetic acid)], and phosphate buffered saline (PBS) (10 mM sodium phosphate, 145 mM NaCl, pH 7.4) were used as buffers.

### Serum and Complement Inhibitors

Normal human serum (NHS) was purchased from Complement Technology and Innovative Research. Factor B-depleted serum and C8-depleted serum were purchased from Complement Technology. The complement inhibitors used were: anti-human C5 (Soliris; eculizumab) (Creative Biolabs), anti-Factor B #1379 inhibitory MoAb (murine IgG1 kappa) (58), Cp20 [a compstatin analog, that binds to and inhibits C3 with higher affinity; generous donation from Dr. John D. Lambris (University of Pennsylvania) and Dr. Daniel Ricklin (University of Basel) (59)], OmCI [*Ornithodoros moubata* C5 complement inhibitor; generous donation from Dr. Susan M. Lea (University of Oxford) (60)], SALO [a specific classical pathway (CP) inhibitor of C1; generous donation from Dr. Jesus G. Valenzuela (National Institutes of Health) (61)].

### Other Reagents

Human properdin was purified as described in the cited reference (13). RH19-20, containing C-terminal domains 19–20 of Factor H, was produced in a *Pichia pastoris* pPICZα expression system, as described previously (56). RH19-20 mutants (R1215Q, W1183L, L1189R, and T1184R) were generously donated by Dr. Andrew P. Herbert (University of Edinburgh) and Dr. David Kavanagh (Newcastle University). Compstatin control peptide (Tocris) was used as the control for Cp20. Factor B was purchased from Complement Technology. Sheep erythrocytes (E_S_) and rabbit erythrocytes (E_R_) were prepared from blood obtained from Rockland Immunochemical. E_S_ coated with C3b (E_S_C3b) were generated as previously described (56). Hemin (Sigma) was dissolved in 50 mM NaOH and 145 mM NaCl and stored at −80°C. The hemin concentration was determined by diluting hemin in H_2_O and measuring the OD at 385 nm (62, 63). The following antibodies were used: monoclonal anti-properdin #1 (anti-P#1, Quidel), goat anti-properdin IgG, mouse anti-human CD59 (IgG2a) (Abcam), mouse IgG1 isotype control (eBioscience), mouse IgG2a isotype control (Abcam), mouse anti-C3c (IgG1) (Quidel), Alexa Fluor 488-conjugated rabbit anti-goat IgG (H+L) (Invitrogen), goat anti-mouse IgG (Fc specific) F(ab')_2_ fragment-FITC (Sigma), Alexa Fluor 488-Goat anti-mouse IgG (H+L) (Invitrogen). The following cell media and dissociation buffer were used: M199 (Gibco), EGM-2 (Lonza), TrypLE (Gibco).

### Production and Purification of Monoclonal Antibodies Against Human Properdin

Anti-properdin hybridoma cell lines were developed by Genscript USA Inc., using our purified human properdin. The antibodies (3A3E1, 6E11A4, 1G6D2, and 6E9E6) were purified by Protein G chromatography and the isotype was determined using a mouse MoAb isotyping test kit (Abd Serotec).

### Western Blotting to Evaluate the Ability of the Purified Monoclonal Antibodies to Bind to Reduced and Non-reduced Properdin

10 ng of non-reduced or reduced properdin were run on SDS gel and subsequently transferred to an activated PVDF membrane. The membrane was blocked with PBS + 5% milk overnight at 4°C. Anti-properdin MoAbs 3A3E1, 6E11A4, 1G6D2, and 6E9E6 and purified mouse IgG1 isotype control were added to the membrane at a concentration of 1 μg/mL and incubated for 1 h at room temperature. Binding of the antibodies to non-reduced or reduced properdin was detected with HRP-conjugated goat anti-mouse IgG. The membrane was incubated with SuperSignal West Femto substrate followed by film exposure and development.

### Direct ELISA

Direct enzyme-linked immunosorbent assays (ELISA) were performed to evaluate the ability of purified MoAbs to bind and recognize purified P forms (P_2_, P_3_, P_4_, or unfractionated P). 96 well plates were coated with various concentrations of purified P forms (P_2_, P_3_, P_4_ or unfractionated P) in PBS (0–160 ng/ml) (100 μl/well) and incubated overnight at 4°C. The plate was washed four times with PBS/0.05% Tween (250 μl/well). PBS/3% bovine serum albumin (BSA) (250 μl/well) was added and the plate was incubated for 2 h at 37°C, followed by 2 washes with PBS/0.05% Tween (250 μl/well). Anti-properdin MoAbs 3A3E1, 6E11A4, 1G6D2, 6E9E6 diluted (400 ng/ml) in PBS/1% BSA/0.05% Tween were added (100 μl/well) and the plate was incubated for 1 h at 37°C followed by rabbit anti-mouse IgG-horseradish peroxidase (Abd Serotec) antibody at 1/1,000 (100 μl/well) and incubating the plate for 1 h at 37°C. Next, 2,2′-Azino-bis (3-ethylbenzothiazoline-6-sulfonic acid) peroxidase substrate (ABTS, Sigma) was added (100 μl/well) and the absorbance of each well at 405 nm was measured using a Tecan Infinite M200 spectrophotometer.

### Detection of Properdin Binding to E_S_C3b, in the Presence or Absence of Anti-Properdin MoAbs, by Flow Cytometry

In order to characterize the function of these anti-properdin MoAbs, flow cytometry was used to measure binding of properdin to C3b-coated erythrocytes in the presence or absence of anti-properdin MoAbs. E_S_C3b were suspended in GVB= at approximately 1 × 10^9^ cells/ml. Anti-properdin MoAbs 3A3E1, 6E11A4, 1G6D2, 6E9E6, anti-P#1 (5 μg/ml), or mouse IgG1 isotype control (5 μg/ml), and properdin (1 μg/ml) were added in a total volume of 180 μl and incubated for 15 min at 4°C. 20 μl of the E_S_C3b (2 × 10^6^ cells/tube) was then added to each tube to make up a total volume of 200 μl and incubated for 1 h at 4°C. The erythrocytes were washed twice by adding 800 μl GVB= to each tube and centrifuging the samples at 3,000 g for 3 min at 4°C. The supernatant was discarded. 200 μl of goat anti-properdin IgG (1/250 dilution) was added and incubated for 30 min at 4°C. After washing twice with 800 μl of GVB=, 200 μl of Alexa Fluor 488 rabbit anti-goat IgG (1/400 dilution) was added and the tubes were incubated for 30 min at 4°C. The erythrocytes were washed twice with 800 μl of GVB=, 800 μl of filtered GVB= was added, and the erythrocytes were transferred to BD Vacutainer tubes. Properdin binding to E_S_C3b was detected by acquiring 10,000 events of the erythrocyte population by flow cytometry (BD FACSCalibur). FlowJo 10.6 was used to determine properdin-associated geometric mean fluorescence intensities (GMFI).

### AP-Mediated Hemolytic Assays

#### AP-Mediated Hemolysis of E_R_ in the Presence or Absence of Anti-Properdin MoAbs

1.2 × 10^7^ E_R_ were mixed with GVB= and the following reagents at the indicated final concentrations: 2.5 mM MgEGTA or 10 mM EDTA, enough NHS to produce ~60% hemolysis (6.5% from Complement Technology or 8% NHS from Innovative Research; NHS used henceforth is from Innovative Research unless specified), and anti-properdin MoAbs 3A3E1, 6E11A4, 1G6D2, 6E9E6, or anti-P#1 (0-16 μg/ml), in a total 100 μl volume. Next, the mix was incubated for 20 min at 37°C, mixing the tubes every 5 min. The tubes were then placed on ice and 400 μl of cold GVBE was added to each tube to stop the reaction. The tubes were spun at 1,000 g for 2 min at 4°C. The absorbance of 200 μl of each supernatant was measured in a microtiter plate at 414 nm. The % of hemolysis was calculated using the formula: [(A414—background A414 in the presence of EDTA) / (maximum A414 determined by water lysis—background A414 in the presence of EDTA)] × 100. The relative % of hemolysis was calculated by dividing % hemolysis at an inhibitor dose by % hemolysis at 0 nM inhibitor dose and graphed. The IC_50_ value was calculated by determining the concentration of MoAb required for 50% inhibition of hemolysis.

#### AP-Mediated Hemolysis of E_H_ With rH19-20 and Anti-CD59 (PNH-Like *in vitro* Model) in the Presence of Complement Inhibitors

Assays were carried out with E_H_ on which CD59 was inhibited with MoAb anti-CD59, as previously described (56), with modifications. Briefly, 5 × 10^6^ E_S_ were mixed with GVB= and the following reagents at the indicated final concentrations: 40% NHS, 5 mM MgEGTA or 10 mM EDTA, 1 μg/ml anti-CD59, and 11.4 μM rH19-20 in the presence of one of the complement inhibitors including 3A3E1 or 6E11A4 (0–53 nM), anti-Factor B (0–1, 830 nM), Cp20 (0–6, 400 nM), eculizumab (0–667 nM), and OmCI (0–5,300 nM), on ice. The mix (24 μl) was immediately incubated for 20 min at 37°C. The reaction was stopped by adding 200 μl cold GVBE. The cells were centrifuged and the OD of supernatant was determined at 414 nm. The relative % of hemolysis was graphed and the IC_90_ value was calculated by determining the concentration of the inhibitor required for 90% inhibition of hemolysis. In addition, the target/inhibitor ratio was calculated by dividing the target protein concentration in plasma (nM) by the IC_90_ of the inhibitor (nM). The concentrations of properdin, Factor B, C3, and C5 in plasma are 14.5 μg/ml (274 nM for a monomer; 91.2 nM for P_3_), 200 μg/ml (2,150 nM), 1.25 mg/ml (6,600 nM), and 75 μg/ml (395 nM), respectively (2).

#### AP-Mediated Hemolytic Assay With RBCs From PNH Patients in the Presence of Complement Inhibitors

Blood from four PNH patients on eculizumab treatment (from John Hopkins University), four PNH patients not under eculizumab treatment [used in (24)] and two healthy adults was collected by venipuncture and the erythrocytes were frozen in liquid nitrogen as described in (64) or −80°C (24). The Institutional Review Board from both the University of Toledo College of Medicine and Life Sciences (for normal donors and RBCs from PNH patients that are not under eculizumab treatment) and John Hopkins University (for eculizumab-treated PNH patients) approved the protocols and written informed consent was obtained from donors, in accordance with the Declaration of Helsinki. PNH RBCs (5 × 10^6^ cells/tube) were mixed with GVB= and the following reagents at the indicated final concentrations: 5 mM MgEGTA or 10 mM EDTA, 40% NHS, 17 μM rH19-20 (for PNH RBCs from patients without eculizumab treatment), one of the complement inhibitors including 3A3E1 or 6E11A4 (0–53 nM), anti-Factor B (0–1,000 nM), Cp20 (0–6,400 nM), eculizumab (0–667 nM), OmCI (0–2,700 nM), or a combination of eculizumab (667 nM) and anti-properdin 6E11A4 (20 nM, IC_50_ dose of the anti-properdin MoAb that inhibits PNH RBCs from lysis). The relative % of hemolysis of PNH RBCs, the IC_90_ of each inhibitor, and the target/inhibitor ratios were assessed as described in section “AP-mediated hemolysis of E_H_ with rH19-20 and anti-CD59 (PNH-like *in vitro* model) in the presence of complement inhibitors”.

#### AP-Mediated Hemolysis of E_S_ With rH19-20 (aHUS-Like *in vitro* Model) in the Presence of Complement Inhibitors

Assays were carried out as described in section “AP-mediated hemolysis of E_H_ with rH19-20 and anti-CD59 (PNH-like *in vitro* model) in the presence of complement inhibitors”, except with E_S_ (instead of E_H_), no anti-CD59 was used, and rH19-20 was used at 1.7 μM, anti-Factor B at 0–500 nM, eculizumab at 0–168 nM, and OmCI at 0–300 nM.

### CD59 and C3 Fragment Profile Analysis of PNH RBCs After Exposure to NHS and rH19-20 in the Presence or Absence of Anti-Properdin MoAbs or Eculizumab

CD59 profile of PNH RBCs and normal RBCs after NHS and rH19-20 exposure (in a hemolytic assay) was determined as previously described (24). Briefly, the hemolytic assay was performed as described in section “AP-mediated hemolysis of E_S_ with rH19-20 (aHUS-Like *in vitro* model) in the presence of complement inhibitors” with the following changes: rH19-20 (20 μM) was added in the presence or absence of one of the complement inhibitors: eculizumab (667 nM), anti-properdin inhibitory MoAb 6E11A4 (53 nM) or non-inhibitory MoAb 6E9E6 (53 nM). The remaining unlysed PNH RBCs were assessed by incubation with anti-CD59 MoAb (Abcam, 5 μg/ml), or mouse IgG2a Isotype control (Abcam, 5 μg/ml), for 45 min at 4°C, followed by incubation with FITC-conjugated rabbit anti-mouse IgG antibodies (Sigma, 1:100 dilution) for 45 min at 4°C, and the cells were then analyzed by flow cytometry (BD FACSCalibur). At least 10,000 events were acquired per sample. FlowJo 10.6 was used to determine CD59-associated GMFI. For C3 fragment analysis, anti-C3b/iC3b/C3dg (Cedarlane, 1 μg/ml) was added in addition to CD59 staining for 45 min at 4°C and the cells were analyzed as described above after gating on the PNH type III cell populations for determining C3-associated GMFI.

### Measurement of Heme-Induced Complement Activation on Human Umbilical Vein Endothelial Cells (HUVECs) After Exposure to NHS in the Presence or Absence of Complement Inhibitors

Heme-induced complement activation on endothelial cells was carried out as described by others (65, 66), with several modifications. Briefly, HUVECs (Passage 5–7, grown in 24-well plates) were stimulated with 500 μl of 100 μM hemin in M199 medium or M199 alone for 30 min at 37°C, washed with M199 and incubated for 30 min at 37°C in NHS (from Complement Technology or Innovative Research) or Factor B-depleted serum (15 or 33% final concentration) in M199 medium, with or without the following reagents [Factor B (54 nM), Cp20 (50 μM), Compstatin control peptide (50 μM), EDTA (20 mM), anti-properdin MoAb (6E11A4, 80 nM), IgG1 Isotype control (80 nM), eculizumab (53 or 80 nM), or SALO (5 μM)], in a total reaction volume of 500 μl. The reaction was stopped with cold PBS and fixed with 0.5% paraformaldehyde for 2.5 min at 4°C. The HUVEC monolayers were stained with mouse MoAb anti-C3c (10 μg/ml) for 30 min at 4°C and subsequently with Alexa Fluor 488-conjugated Goat anti-mouse IgG (H+L) (20 μg/ml) for 30 min at 4°C. The cells were washed and detached with TrypLE for 3 min at 37°C. The dissociation was stopped by adding PBS and cells were centrifuged for 5 min at 200 g at 4°C. Cells were analyzed by flow cytometry (BD FACSCalibur) by acquiring 7,000–10,000 events per sample. FlowJo 10.6 was used to determine C3-associated GMFI.

### Measurement of Complement Activation on HUVECs After Exposure to NHS, When Factor H Regulation Is Impaired, in the Presence or Absence of Complement Inhibitors

Assays were carried out as described in section “Measurement of heme-induced complement activation on human umbilical vein endothelial cells (HUVECs) after exposure to NHS in the presence or absence of complement inhibitors”, except that the hemin- or M199-incubated HUVECs were incubated with 33% NHS with or without 10 μM rH19-20 or aHUS-related Factor H mutants (R1215Q, W1183L, L1189R, and T1184R) in the presence or absence of Cp20 (50 μM), anti-properdin MoAb (6E11A4, 53 nM), or IgG1 Isotype control (53 nM) for 30 min at 37°C.

### Statistics

GraphPad Prism 7.0 was used to analyze data. One-way ANOVA or two-way ANOVA with multiple comparison tests (indicated in figure legends) was used to determine statistical significance between groups. *P* < 0.05 were considered statistically significant.

## Results

### Generation and Characterization of Anti-Human Properdin MoAbs 3A3E1, 6E11A4, 1G6D2, and 6E9E6

**Figure 1:**
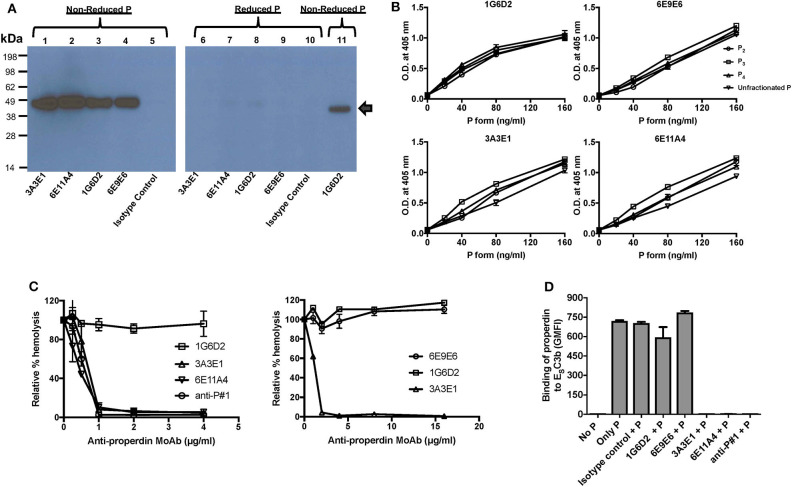
Generation and characterization of anti-properdin MoAbs (3A3E1, 6E11A4, 1G6D2, 6E9E6). **(A)** Anti-properdin MoAbs recognize non-reduced properdin (P) but not reduced properdin (P). 10 ng of non-reduced or reduced unfractionated properdin was detected with anti-properdin MoAbs 3A3E1, 6E11A4, 1G6D2, or 6E9E6, or purified mouse IgG1 isotype control at 1 μg/ml by immuno Western blot. Binding of the antibodies to non-reduced (lane 1–5, 11) or reduced (lane 6–10) properdin is shown. Lane 11 is a positive control which contains 10 ng of purified properdin detected by 1 μg/mL 1G6D2 anti-properdin MoAb under non-reduced conditions. The arrow indicates the properdin monomer size (~50 kDa). **(B)** Anti-properdin MoAbs recognize all properdin forms [P_2_, P_3_, P_4_, or unfractionated properdin (P)] in a direct ELISA. The binding was detected as described in “Materials and Methods.” Results are shown as mean and SD of triplicates. **(C)** 3A3E1 and 6E11A4 inhibit AP-mediated hemolysis of rabbit erythrocytes (E_R_). E_R_ were incubated with NHS in the presence of one of the indicated anti-properdin MoAbs. The hemolytic activity was determined as described in “Materials and Methods” and was expressed as a relative percentage (%) of hemolysis. Results on the left are shown as mean and SD of duplicate observations. Results on the right are shown as mean and SD of duplicate observations for 6E9E6 and single observations for controls 3A3E1 and 1G6D2. **(D)** 3A3E1 and 6E11A4 inhibit the ability of properdin to bind to E_S_C3b. Each anti-properdin MoAb (1G6D2, 6E9E6, 3A3E1, 6E11A4, or anti-P#1) or isotype control was incubated with properdin (P) and E_S_C3b. The binding of properdin to E_S_C3b was determined as described in “Materials and Methods.” “No P” was a negative control where no properdin was added, and “Only P” was a positive control where no inhibition antibody was added. The graph is a representative of three independent experiments and is shown as mean and SD of duplicates. The data were analyzed by one-way ANOVA with Dunnett's multiple comparisons against “No P”; *p* < 0.0001 in “Only P,” “Isotype control+P,” “1G6D2+P,” “6E9E6+P” groups.

The four anti-human properdin clones were selected based on their ability to strongly recognize properdin when evaluated in a direct ELISA and these antibodies were purified by protein G chromatography and determined to be IgG1 kappa isotype (data not shown). The purified anti-properdin MoAbs recognize only non-reduced properdin, as evaluated by immuno western blot (Figure 1A), and recognize human, baboon, rabbit, and canine properdin, but not mouse, rat, feline or ferret properdin (data not shown). In addition, to determine whether the MoAbs recognize distinct epitopes, competition ELISAs were carried out where each anti-properdin antibody was biotinylated and mixed with each of the other unlabeled anti-properdin antibody clones, followed by incubation with properdin-coated ELISA plates, and binding of the biotinylated antibody to properdin was measured. 6E9E6 and 1G6D2 did not compete with each other for binding to properdin (Figures S1A,B) and thus recognize distinct epitopes on properdin, while 3A3E1 and 6E11A4 did compete for binding to properdin in the competitive ELISA (Figures S1C,D), indicating they recognize the same or overlapping epitopes. Because properdin oligomerization is associated with the function of properdin [reviewed in (14, 16)], we sought to determine whether the antibodies could distinguish between dimers (P_2_), trimers (P_3_), and tetramers (P_4_) of properdin using purified properdin oligomers in direct ELISAs. Figure 1B indicates that the MoAbs recognize the different oligomers of properdin to the same extent and cannot distinguish between them.

#### The Anti-Properdin MoAbs Inhibit Properdin-Mediated Stabilization of the C3bBb Convertase of the AP by Inhibiting the Binding of Properdin to C3b

In order to determine whether the antibodies inhibit the ability of properdin to stabilize the C3bBb convertases, an AP hemolysis assay was performed using rabbit erythrocytes. Anti-properdin antibodies 6E11A4 or 3A3E1 inhibited the hemolysis of rabbit erythrocytes in a dose-dependent manner, whereas 1G6D2 and 6E9E6 did not inhibit cell lysis, even when tested at 16 μg/ml (Figure 1C). Commercial anti-P#1 was used as a positive control for inhibition. The half maximal inhibitory concentration (IC_50_) was 0.5 μg/ml for 6E11A4, was 0.6 μg/ml for anti-P#1 and was 0.7 μg/ml for 3A3E1. Because properdin needs to bind to C3b in order to stabilize the convertase, the ability of the antibodies to inhibit the binding of properdin to C3b was tested by flow cytometry. Figure 1D shows that 3A3E1 and 6E11A4 completely impaired the binding of properdin to C3b-coated erythrocytes (E_S_C3b), whereas 1G6D2 and 6E9E6 did not.

### Determination of the Role of Properdin in Promoting Complement-Mediated Lysis of PNH RBCs *in vitro*

#### Inhibition of Properdin Prevents Complement-Mediated Lysis of “PNH-like” Human Erythrocytes (E_H_), and Does so More Effectively Than Other Complement Inhibitors

**Figure 2:**
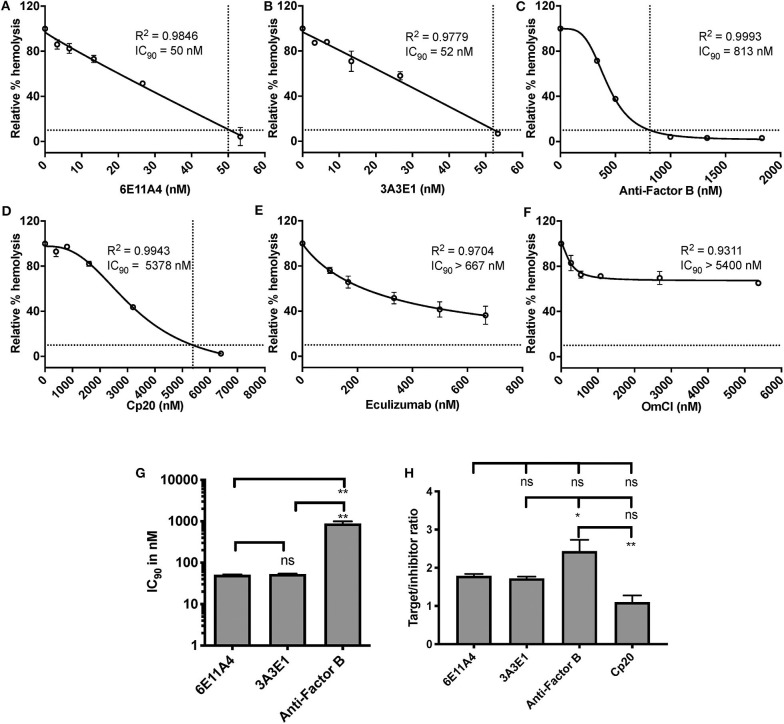
Efficiency of inhibiting properdin as compared to other complement inhibitors on preventing lysis of “PNH-like” RBCs. **(A–F)** Determination of IC_90_ values of complement inhibitors for inhibiting complement-mediated lysis of “PNH-like” human erythrocytes (E_H_) and the data are shown as mean and SD of duplicate observations. E_H_ were mixed with the following reagents at the indicated final concentrations: 40% NHS, 5 mM MgEGTA or 10 mM EDTA, 11.4 μM rH19-20, 1 μg/ml anti-CD59, and one of the complement inhibitors 6E11A4 **(A)**, 3A3E1 **(B)**, anti-Factor B **(C)**, Cp20 **(D)**, eculizumab **(E)**, and OmCI **(F)** at the concentrations indicated in the graphs. The relative % hemolysis and IC_90_ of each inhibitor was determined as described in “Materials and Methods” and the dotted line is where the inhibition reaches to 90%. IC_90_ values obtained in **(A–F)** were graphed in **(G)** and combined from two independent experiments with duplicates for each inhibitor, shown as mean and SD. IC_90_ values in **(G)** were divided by plasma concentration (nM) of each inhibitor to evaluate target/inhibitor ratio in **(H)**. Properdin was considered as a trimer for calculation and eculizumab and OmCI results were not included because no IC_90_ values could be determined. The data were analyzed by one-way ANOVA with Tukey's multiple comparison test; **p < 0.01, *p < 0.05, *p* > 0.05 ns.

Using the characterized inhibitory anti-properdin MoAbs, we explored the role of properdin on promoting complement-mediated lysis of RBCs with complement regulation defects, such as those RBCs in PNH patients. We first used E_H_ where CD59 has been blocked using an inhibitory anti-CD59 antibody (“PNH-like” cells) and rH19-20 was added as a way of increasing the overall detectable serum-induced lysis by removing Factor H protection, which obviates the need to acidify the serum in order to achieve the same outcome, as we have previously described (24). Using this assay, we assessed the effect of blocking properdin with anti-properdin MoAbs as compared with blocking other complement components, including (a) anti-Factor B, which inhibits the AP convertase formation by inhibiting binding of Factor B to C3b; (b) Cp20, which binds C3 and interferes with C3 cleavage and thus prevents convertase formation; and (c) eculizumab and OmCI, which inhibit C5 cleavage and thus the formation of C5a and MAC. Each inhibitor was added in increasing concentrations to the assay, and the IC_90_ for each inhibitor was determined (Figures 2A–F). Figure 2G shows the combined IC_90_ values for the different inhibitors. The anti-properdin MoAbs 6E11A4 and 3A3E1 gave an IC_90_ of 51 and 53 nM, respectively. The IC_90_ obtained for anti-Factor B was 890 nM, approximately 17-fold higher than the IC_90_ for the anti-properdin MoAbs. Cp20 gave an IC_90_ value of ~6,058 nM, which was ~117-fold higher than the anti-properdin MoAbs (not shown). No IC_90_ value could be obtained for eculizumab and OmCI, even when tested at 667 and 5,400 nM, respectively. In order to take into account the variations in plasma concentrations of each targeted complement protein, target/inhibitor ratios were calculated for each inhibitor by dividing the nM plasma concentrations of the target by the average IC_90_ values, as described in “Materials and Methods”. The ratio for anti-properdin MoAbs was 5.2–5.4 (for monomeric properdin; not shown) or 1.7–1.8 (for the trimer form of properdin, P_3_, the predominant form in plasma), for anti-Factor B was 2.4, and for Cp20 was 1.1 (Figure 2H). This indicates that inhibiting properdin, if considered as a trimer, had similar target/inhibitor ratio as the other inhibitors tested. The ratio for eculizumab and OmCI could not be determined due to their lack of IC_90_ values. Overall, the data show that properdin is essential for promoting “PNH-like” RBC lysis and, based on the IC_90_ values and target/inhibitor ratios, blocking properdin with anti-properdin MoAbs was at least as efficient as blocking other complement components, i.e., Factor B, C3, at inhibiting AP-mediated lysis of E_H_ that lack CD59 cell surface protection, while being more effective than C5 inhibition (Figure 2).

#### Inhibition of Properdin Inhibits the Lysis of RBCs Derived From PNH Patients by Protecting PNH Type II and III Cells From Lysis

**Figure 3:**
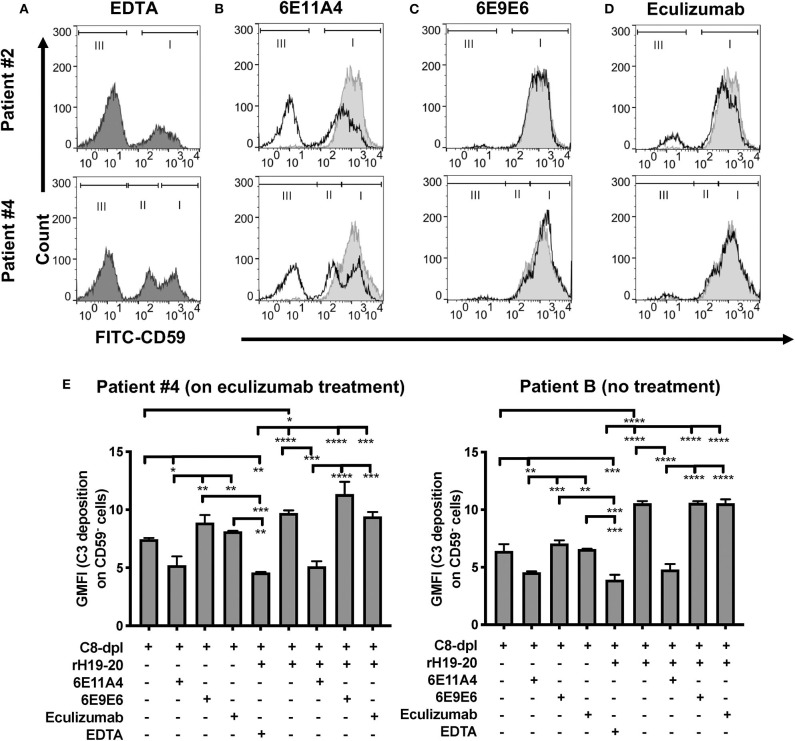
Inhibition of properdin protects PNH type II and III cells from complement opsonization and lysis. RBCs from two PNH patients under eculizumab treatment and one PNH patient without eculizumab treatment were mixed with the following reagents at the indicated final concentrations: **(A)** 40% NHS + 10 mM EDTA + 20 μM rH19-20 (dark gray shaded histograms; original PNH RBCs population distribution control), or **(B–D)** NHS + 5 mM MgEGTA + 20 μM rH19-20, in the absence (light gray histograms) or presence (black line) of one the complement inhibitors [**(B)** anti-properdin inhibitory MoAb 6E11A4 (53 nM), **(C)** non-inhibitory MoAb 6E9E6 (53 nM), or **(D)** eculizumab (667 nM)], or **(E)** 40% C8-depleted serum (C8-dpl) + 5 mM Mg-EGTA in the presence or absence of 1 μM rH19-20, 53 nM 6E11A4, 53 nM 6E9E6, or 667 nM eculizumab. 10 mM EDTA was used as a negative control. CD59 and C3 fragments analysis of the remaining unlysed cells was assessed as described in “Materials and Methods.” The population of normal RBCs, type II and type III PNH RBCs, are indicated by the markers I, II and III, respectively **(A–D)**. The graphs are representative of two independent experiments with duplicate observations. The data were analyzed by one-way ANOVA with Tukey's multiple comparison test. Only relevant statistics were shown in **(E)**; ****p < 0.0001, ***p < 0.005, **p < 0.01, *p < 0.05, *p* > 0.05 ns.

With RBC samples from PNH patients who are under eculizumab treatment, we next determined which types of PNH cells are protected from complement-mediated lysis when properdin is inhibited. We have previously shown that PNH type II and III cells become highly susceptible to complement-mediated lysis when Factor H protection is removed via the addition of rH19-20 to serum (24), which is similar to what occurs in serum that has been acidified to pH 6.4 (67, 68). Figure 3A shows the CD59^+^ distribution of the RBCs from two patients when exposed to serum in the absence of complement activity (EDTA control). When the RBCs are treated with NHS in the presence of MgEGTA and rH19-20, the PNH type II and III cells are lysed, leaving the normal RBCs intact, as expected (Figures 3B–D, light gray shaded histograms). Figures 3B,D (black lines vs. light gray shaded histograms) show that, unlike eculizumab, which partially prevented the rH19-20-mediated lysis of PNH RBCs (Figure 3D), inhibition of properdin via MoAb 6E11A4 completely protected PNH RBCs from AP-mediated lysis, thus restoring the PNH type II and III RBC population to a similar distribution as the EDTA group (Figures 3A,B). The non-inhibitory anti-properdin MoAb 6E9E6 did not prevent PNH type II and III RBCs from AP-mediated lysis (Figure 3C). Moreover, anti-properdin MoAb 6E11A4 prevents C3 fragment deposition on PNH RBCs that lack CD55/CD59 protection, while eculizumab and 6E9E6 cannot (Figure 3E). This indicates that blocking properdin stabilization function protected PNH type II and III RBC from complement activation and lysis when Factor H protection is removed and was more efficient than eculizumab.

#### Inhibition of Properdin Inhibits Lysis of PNH Patient RBCs More Effectively Than Other Complement Inhibitors

**Figure 4:**
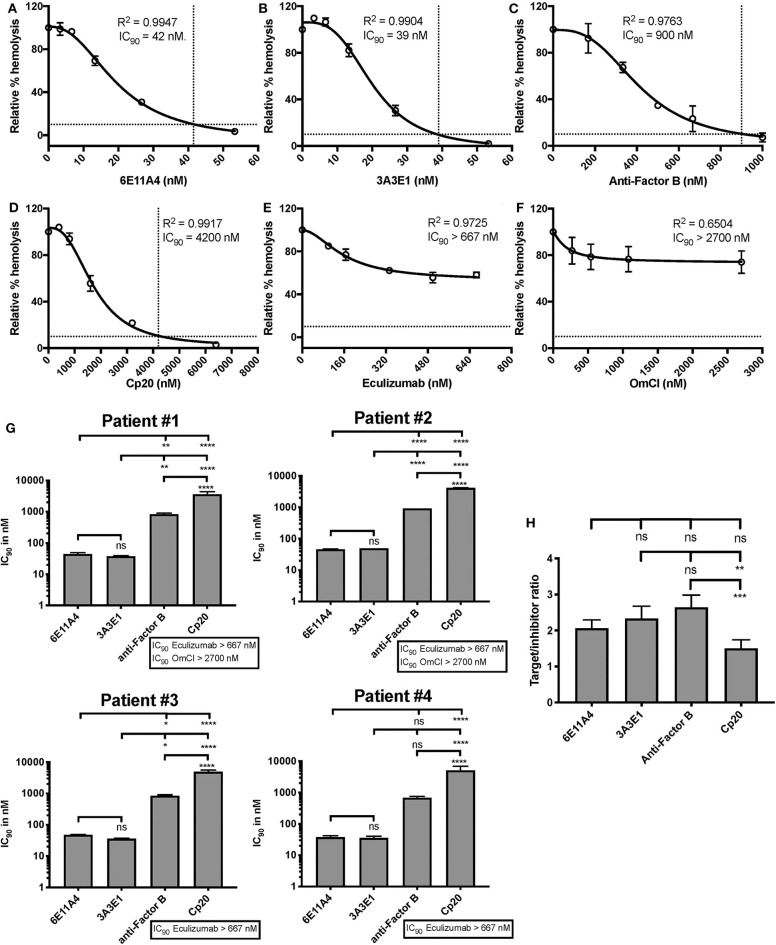
Efficiency of inhibiting properdin as compared to other complement inhibitors on preventing lysis of PNH RBCs. **(A–F)** Determination of IC_90_ values of complement inhibitors for inhibiting complement-mediated lysis of RBCs from PNH patients. PNH patient #1 results are shown as a representative of data from four PNH patients under eculizumab treatment, whose RBCs were mixed with the following reagents at the indicated final concentrations: 40% NHS, 5 mM MgEGTA or 10 mM EDTA, and one of the complement inhibitors 6E11A4 **(A)**, 3A3E1 **(B)**, anti-Factor B **(C)**, Cp20 **(D)**, eculizumab **(E)**, and OmCI **(F)** at the concentrations indicated in the graphs. The relative % hemolysis was determined as described in “Materials and Methods” and the dotted line is where the inhibition reaches to 90%. The IC_90_ values for four PNH patients, obtained as in **(A–F)**, were graphed in **(G)**. OmCI data was from two patients (#1 and #2) due to limited supplies. Results are representative of at least two independent experiments with duplicates and are shown as mean and SD. IC_90_ values in **(G)** were divided by plasma concentration (nM) of each inhibitor to evaluate target/inhibitor ratio in **(H)**. Properdin was considered as a trimer for calculation and eculizumab and OmCI results were not included because no IC_90_ values could be determined. The data were analyzed by one-way ANOVA with Tukey's multiple comparison test; ****p < 0.0001, ***p < 0.001, **p < 0.01, *p < 0.05, *p* > 0.05 ns.

**Figure 5:**
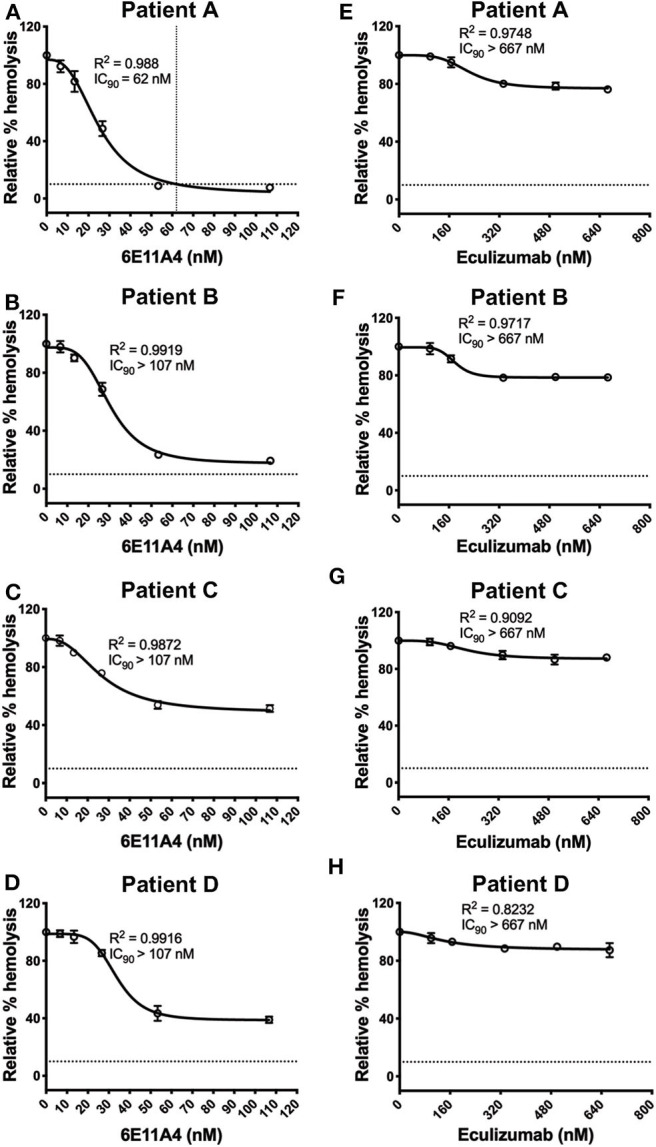
Blocking properdin inhibits complement-mediated lysis of PNH RBCs that has not undergone eculizumab treatment. **(A–H)** Determination of IC_90_ values of complement inhibitors for inhibiting complement-mediated lysis of RBCs from PNH patients **(A–D)**. PNH RBCs were mixed with the following reagents at the indicated final concentrations: 17 μM rH19-20, 40% NHS, 5 mM MgEGTA or 10 mM EDTA, and one of the complement inhibitors 6E11A4 **(A–D)**, eculizumab **(E–H)** at the concentrations indicated in the graphs. The relative % hemolysis was determined as described in “Materials and Methods” and the dotted line is where the inhibition reaches to 90%. Results are representative of average values from two independent experiments with duplicates and are shown as mean and SD.

**Figure 6:**
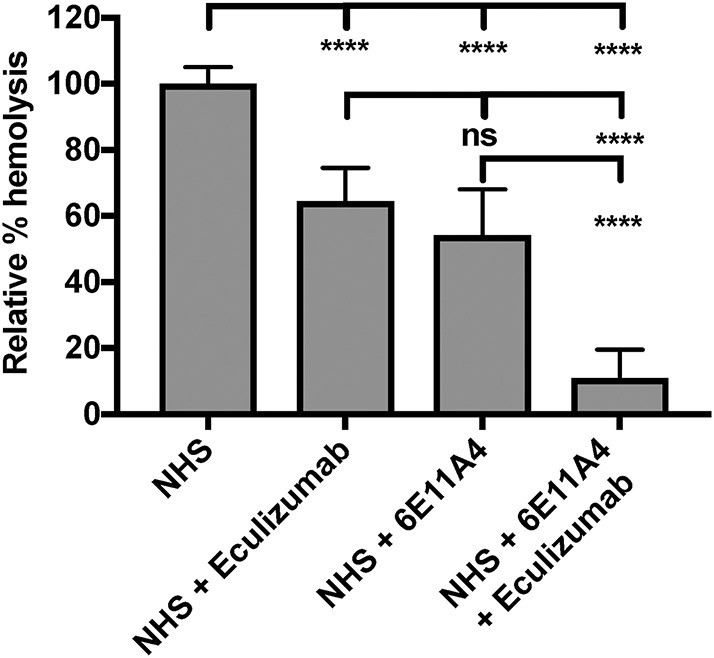
Combination of eculizumab and anti-properdin MoAb 6E11A4 overcomes the residual hemolysis of C5 inhibition. PNH RBCs were mixed with the following reagents at the indicated final concentrations: 40% NHS, 5 mM MgEGTA or 10 mM EDTA, in the presence of 667 nM eculizumab, or 20 nM (IC_50_ dose as shown in Figure 4A) 6E11A4, or the combination of 667 nM eculizumab and 20 nM 6E11A4. The hemolytic activity was determined as described in “Materials and Methods” and was expressed as a % hemolysis relative to “NHS.” Results are combined from three independent experiments (with duplicates) and are shown as mean and SD. The data were analyzed by one-way ANOVA with Tukey's multiple comparison test; ****p < 0.0001, *p* > 0.05 ns.

The PNH RBC protection obtained by blockage of properdin was compared to that of inhibiting other complement components (listed in Figure 2). RBCs derived from four PNH patients that are under eculizumab treatment had 35–65% basal hemolysis in the presence of 40% NHS and MgEGTA (data not shown), likely due to the high percentage (>70%) of type II and III RBCs among the total RBC population (Figure S2). Figures 4A–F show the data from PNH patient #1 as representative of the screening of all complement inhibitors in their ability to protect PNH patient RBC from complement-mediated lysis. Blocking properdin, Factor B or C3 completely protected PNH RBCs that are from patients who are under eculizumab treatment from complement-mediated hemolysis (Figures 4A–D). The IC_90_ values for anti-properdin MoAbs 6E11A4 and 3A3E1 in these four patients ranged from 38–48 nM and 36–50 nM, respectively, which were at least 18-fold, 81-fold lower than that of anti-Factor B (with IC_90_ 688–920 nM) and Cp20 (with IC_90_ 3,650–5,150 nM), respectively (Figure 4G). The target/inhibitor ratios for the anti-properdin MoAbs 6E11A4 and 3A3E1 were 5.5–7.7 (for the monomer; not shown) or 1.8–2.6 (for P_3_, the main form in plasma), for anti-Factor B was 2.3–3.1, and for Cp20 was 1.3–1.8 (Figure 4H). This indicates that inhibiting properdin, if considered as a trimer, had similar target/inhibitor ratio as the other inhibitors tested. However, eculizumab and OmCI, the two C5 inhibitors, failed to inhibit PNH patient RBC lysis completely and reached a plateau of background hemolysis above 50% (Figures 4E,F). The IC_90_ for eculizumab and OmCI in the four patient RBCs was above 667 nM (above 13-fold more than the anti-properdin MoAbs IC_90_) and 2,700 nM (above 54-fold higher than anti-properdin MoAbs IC_90_), respectively (Figure 4G), indicating that blocking C5 was inefficient for protecting these PNH RBCs. PNH RBCs from patients who are not treated with eculizumab were also used to test whether blocking properdin would protect these cells. Instead of using acidified serum, rH19-20 was used in the assay, as described previously (24), to increase the sensitivity required for assessing complement-mediated hemolysis. Blocking properdin inhibits the lysis more than 50% in all untreated patients, and IC_90_ could be determined in patient A (Figures 5A–D), while eculizumab had a marginal protective effect (Figures 5E–H). The inefficiency of eculizumab on protecting these cells is intrinsic to C5 inhibition as a similar effect is observed using another C5 inhibitor, OmCI (data not shown). Given that eculizumab, the current treatment for PNH, resulted in incomplete inhibition of PNH RBC lysis (Figures 4E, 5E–H), we next explored the consequences of using the combination of anti-properdin MoAbs and eculizumab in our *in vitro* model (Figure 6). Anti-properdin MoAb 6E11A4 alone, at the IC_50_ dose (Figure 4A), reduced the hemolysis by ~50% and eculizumab alone, at the maximum plateau concentration (Figure 4E; 667 nM), reduced hemolysis by ~40% (Figure 6), as expected. The combination of anti-properdin MoAb and eculizumab significantly reduced the residual hemolysis caused by eculizumab to 10%, and the inhibition effect was additive (Figure 6).

### Determination of the Role of Properdin in Promoting Complement-Mediated Damage of RBCs and Endothelial Cells in aHUS *in vitro*

#### Inhibition of Properdin Prevents Lysis of Sheep Erythrocytes (E_S_) Under an “aHUS-Like” Condition

AHUS is another disease of complement dysregulation that is characterized by hemolysis, which is traditionally explained by mechanical hemolysis as a result of RBCs passing through narrowed microvasculature due to microthrombi formation, and by complement-mediated lysis due to lack complement regulation on RBCs [reviewed in (69)]. Because mutations in domains 19 and 20 of Factor H are highly associated with aHUS (7), we used recombinant protein rH19-20 that competitively inhibits Factor H-mediated cell surface protection on E_S_, which are normally resistant to AP, resulting in RBC lysis. The inability of Factor H to bind to the RBC surface (to protect it) mimics what happens when aHUS patients have mutations in the C-terminus of Factor H (56).

**Figure 7:**
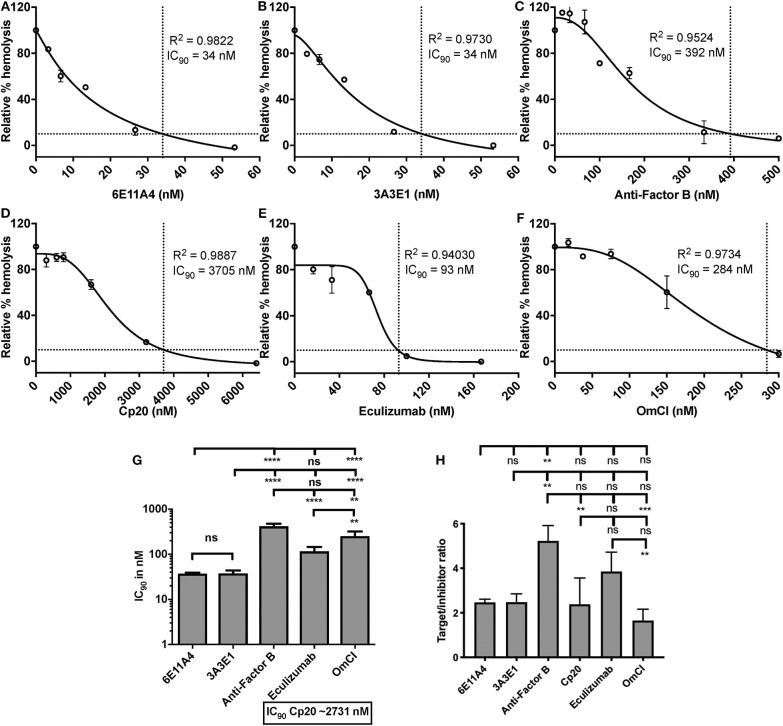
Efficiency of inhibiting properdin as compared to other complement inhibitors on preventing lysis of sheep erythrocytes (E_S_) under an “aHUS-like” condition. **(A–F)** Determination of IC_90_ values of complement inhibitors on preventing lysis of “aHUS-like” sheep erythrocytes (E_S_), and the data are shown as mean and SD of duplicate observations. E_S_ were mixed with the following reagents at the indicated final concentrations: 40% NHS, 1.7 μM rH19-20, 5 mM MgEGTA or 10 mM EDTA, and one of the complement inhibitors 6E11A4 **(A)**, 3A3E1 **(B)**, anti-Factor B **(C)**, Cp20 **(D)**, eculizumab **(E)**, and OmCI **(F)** at the concentrations indicated in the graphs. The relative % hemolysis and IC_90_ of each inhibitor was determined as described in “Materials and Methods” and the dotted line is where the inhibition reaches to 90%. The IC_90_ values obtained as in **(A–F)** were graphed in **(G)** and are combined from more than three independent experiments with duplicates for each inhibitor and are shown as mean and SD. IC_90_ values in **(G)** were divided by plasma concentration (nM) of each inhibitor to evaluate target/inhibitor ratio in **(H)**. Properdin was considered as a trimer for calculation. The data were analyzed by one-way ANOVA with Tukey's multiple comparison test; ****p < 0.0001, ***p < 0.005, **p < 0.01, *p* > 0.05 ns.

Using the *in vitro* assay, the ability of inhibiting properdin to reduce AP activity (mediated by lack of Factor H protection) was tested and compared to inhibiting other complement components (inhibitors listed in Figure 2). Each complement inhibitor was added in increasing concentrations to the E_S_ cells in the presence of NHS and rH19-20, and the IC_90_ for each inhibitor was determined (Figures 7A–F). Figure 7G data indicates that the anti-properdin MoAbs 6E11A4 and 3A3E1 gave IC_90_ values between 34 and 45 nM, and blocking properdin using anti-properdin MoAbs had 3–86-fold lower IC_90_ values for inhibiting AP-mediated lysis of “aHUS-like” RBCs than the other complement inhibitors tested (IC_90_: Factor B = 416 nM; eculizumab = 107 nM; OmCI = 252 nM; Cp20 = 3,174 nM). The target/inhibitor ratios for anti-properdin MoAbs were 7.4 (for monomeric properdin; not shown) or 2.5 (for P_3_, the predominant form in plasma), for anti-Factor B was 5.2, for Cp20 was 2.4, for eculizumab was 3.9, and for OmCI was 1.7 (Figure 7H), indicating that inhibiting properdin trimers was as effective as blocking C5 and less effective than blocking Factor B.

#### Inhibition of Properdin Reduces Complement Activation on Heme-Exposed Endothelial Cells

**Figure 8:**
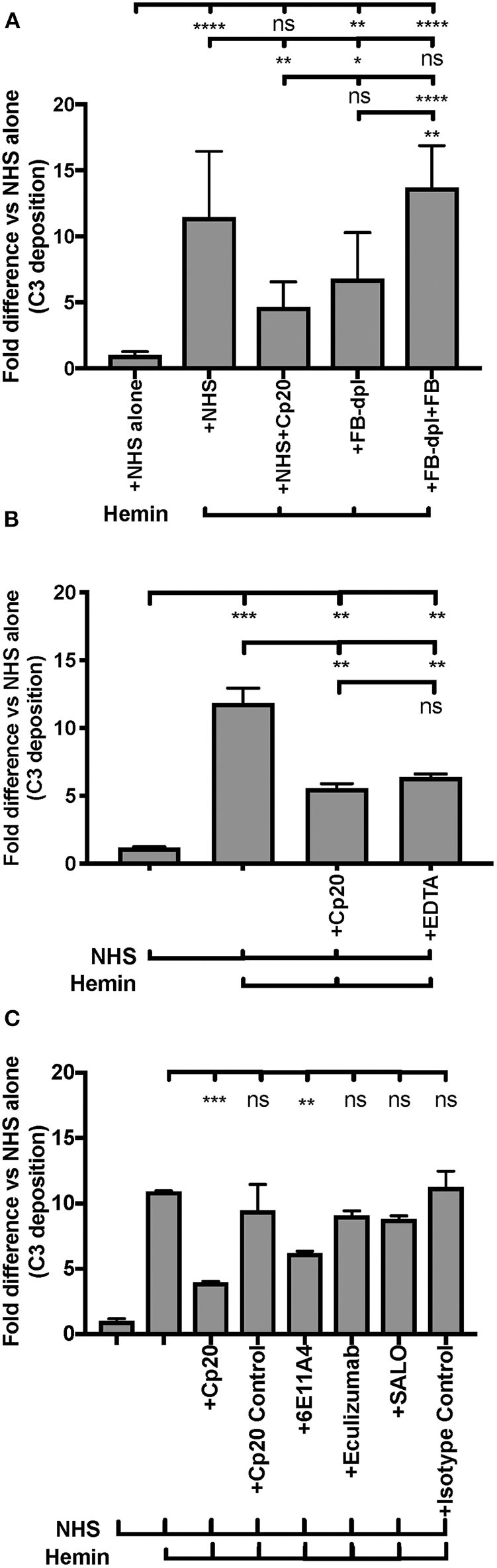
Inhibition of properdin protects endothelial cells from heme-induced complement activation. **(A,B)** Complement activation on heme-treated endothelial cells is alternative-pathway dependent. HUVECs were stimulated with M199 media or 100 μM hemin, washed and incubated with NHS or Factor B-depleted serum (FB-dpl) (15% final), in the presence or absence of Cp20 (50 μM) or EDTA (20 mM). 54 nM Factor B (FB) was used to restore Factor B-depleted serum activity. **(C)** Anti-properdin MoAb protects heme-treated HUVECs from C3 fragment deposition. HUVECs were stimulated with 100 μM hemin (or M199), NHS (33% final) and one of the following reagents at the indicated final concentrations: Cp20 (50 μM), Compstatin control peptide (“Cp20 Control,” 50 μM), anti-properdin MoAb 6E11A4 (80 nM), IgG1 isotype control (80 nM), eculizumab (53 nM or 80 nM) or SALO (5 μM). **(A–C)** C3 deposition (GMFI) was determined as described in “Materials and Methods” and “Fold difference” was calculated by dividing GMFI of each group by GMFI of NHS alone group. **(A)** is a combination of four independent experiments (each with duplicates), **(B,C)** are representative of four and two independent experiments, respectively, with duplicates. The data was analyzed by one-way ANOVA with Tukey's multiple comparison test. ****p < 0.0001, ***p < 0.001, **p < 0.01, *p < 0.05, *p* > 0.05 ns. Statistical analysis between NHS+heme group and each inhibitor group is shown in **(C)**.

Endothelial injury is another typical feature of aHUS patients. The development of aHUS could be triggered by an endothelial cell insult and promoted by abnormal complement regulation. In normal conditions, hemoglobin and heme that are released during hemolysis, are scavenged by haptoglobin and hemopexin, respectively. However, cell-free heme can be found at high concentrations in various disorders that are characterized by excessive hemolysis (70, 71) and can activate the AP of complement on the endothelial cell surface, promoting C3b deposition and MAC formation, as well as induce C3a and C5a generation in the fluid phase of blood (66). These complement activation products have the potential to promote a pro-thrombotic status [reviewed in (72, 73)]. An *in vitro* endothelial assay (66) was adapted into our study to evaluate if properdin modulates heme-induced complement activation on human primary endothelial cells, via measuring deposits of C3 activation products. Figure 8A shows that heme induced 5 to 15-fold higher C3b deposition on HUVECs, vs. NHS alone, and the activation was inhibited 60% by the C3 inhibitor Cp20. Inhibition of complement activity by Cp20 was complete as determined by comparison to a 20 mM EDTA control, where inhibition was also 60% (Figure 8B). Factor B-depleted serum, which lacks AP activity, reduced C3 fragment deposition to a similar level as that of using Cp20 (Figure 8A), while addition of Factor B to Factor B-depleted serum restored C3 fragment deposition induced by heme to a similar level as NHS (Figure 8A), indicating that the heme-induced complement activation is AP-dependent, as described previously by others (66).

Blocking properdin using anti-properdin MoAb 6E11A4 significantly inhibited C3 fragment deposition on heme-treated HUVECs by 40% and eculizumab did not reduce C3 fragment deposition (Figure 8C) as compared with NHS+heme alone group. In addition, SALO, a classical pathway inhibitor (61), did not inhibit C3 fragment deposition on NHS+heme-treated HUVECs (Figure 8C), indicating that the classical pathway does not contribute to complement activation on heme-exposed endothelial cells, confirming Figure 8A data. Altogether, the data indicate that inhibition of properdin function significantly reduced heme-induced complement activation on endothelial cells.

#### Standardization of an aHUS-Like C3b Deposition Assay on Human Endothelial Cells Using Heme and rH19-20 aHUS Mutants

**Figure 9:**
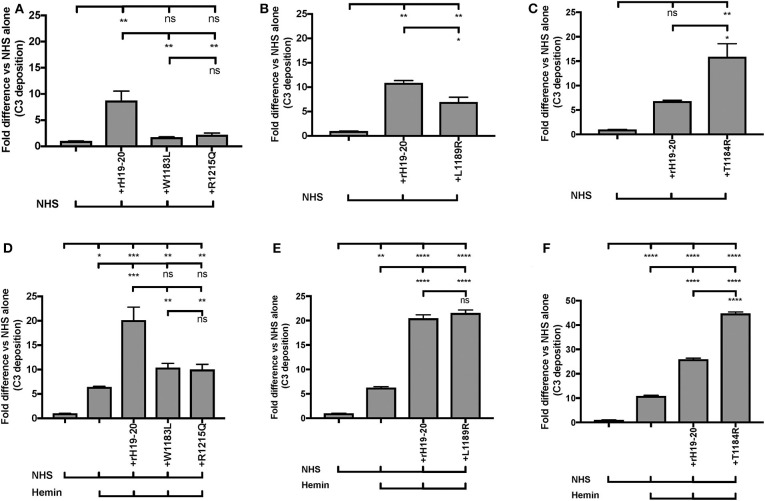
RH19-20 mutants have various effects relative to wild type rH19-20 on affecting complement activation on endothelial cells in the presence or absence of heme. **(A–F)** HUVECs were stimulated with M199 or 100 μM hemin, washed and incubated with NHS (33% final) with or without rH19-20 (10 μM) or aHUS-related mutants (R1215Q, W1183L, L1189R or T1184R, 10 μM). C3 deposition (GMFI) was determined as described in “Materials and Methods.” Representative results are shown as “Fold difference” and graphed as the mean and SD of duplicates. The data were analyzed by one-way ANOVA with Tukey's multiple comparison test; ****p < 0.0001, ***p < 0.001, **p < 0.01, *p < 0.05, *p* > 0.05 ns.

AHUS-related mutations in the C-terminus of Factor H affect its cell surface function and cause pathological events on RBCs (57), platelets (18), neutrophils (18), and endothelial cells (74–76). We thus sought to establish an “aHUS-like” condition on endothelial cells where Factor H protection is impaired and heme is present. For this we assessed how aHUS-related mutations in Factor H domain 19-20 affect complement activation on endothelial cells when exposed to NHS only, as compared to wild type rH19-20. Exposure of the endothelial cells to wild type rH19-20 increased C3 fragment deposition by 7 to 11-fold as compared with NHS alone (Figures 9A–C). Four rH19-20 constructs containing mutations were tested. These mutations have highly impaired (R1215Q and W1183L), intermediately impaired (L1189R) or minimally impaired (T1184R) ability to compete with Factor H and induce complement activation on platelet, neutrophil, and endothelial cell surfaces, as compared to wild type rH19-20 (18, 57, 76). R1215Q and W1183L completely lost their ability to compete with Factor H resulting in no increase in C3 fragment deposition as compared to NHS alone (Figure 9A). L1189R was impaired by 40% in its ability to induce C3 deposition as compared with wild type rH19-20 (Figure 9B). T1184R was not impaired in its ability to induce complement activation (Figure 9C), and increased C3 deposition by 2-fold as compared with wild type rH19-20, in agreement with our previous observation on platelets (18). Because excessive hemolysis is one of the hallmarks of aHUS, we next added heme, in addition to the NHS and rH19-20 wild type or mutants, as a way of establishing an aHUS-like *in vitro* assay. Figures 9D–F shows that exposure of heme alone to endothelial cells increased 6–11-fold complement activation as compared with NHS, as observed in Figure 8. Addition of rH19-20 significantly enhanced heme-induced complement activation by 2–3-fold (Figures 9D–F). Figure 9D shows that the highly impaired mutations (R1215Q and W1183L) had similar impairment trend as compared to that in the absence of heme (Figure 9A). L1189R mutant was no longer intermediately impaired, as it induced the same level of C3 deposition as compared with wild type rH19-20, in the presence of heme (Figure 9E). The minimally impaired mutant T1184R induced 2-fold more C3 deposition vs. the wild type rH19-20 in the presence of heme (Figure 9F), comparable to that observed in the absence of heme (Figure 9C). Collectively, Figure 9 shows that lack of Factor H cell-surface protection led to complement activation on endothelial cells and pre-exposure to heme significantly exacerbated complement activation when endothelial cells are not protected by Factor H. In addition, this aHUS-like C3b deposition assay was able to detect differences between the level of impairment between the rH19-20 mutants tested, similar to what we have observed previously for RBCs, neutrophils and platelets (18, 57).

#### Inhibition of Properdin Reduces Complement Activation on Heme-Exposed Endothelial Cells That Lack Factor H Protection

**Figure 10:**
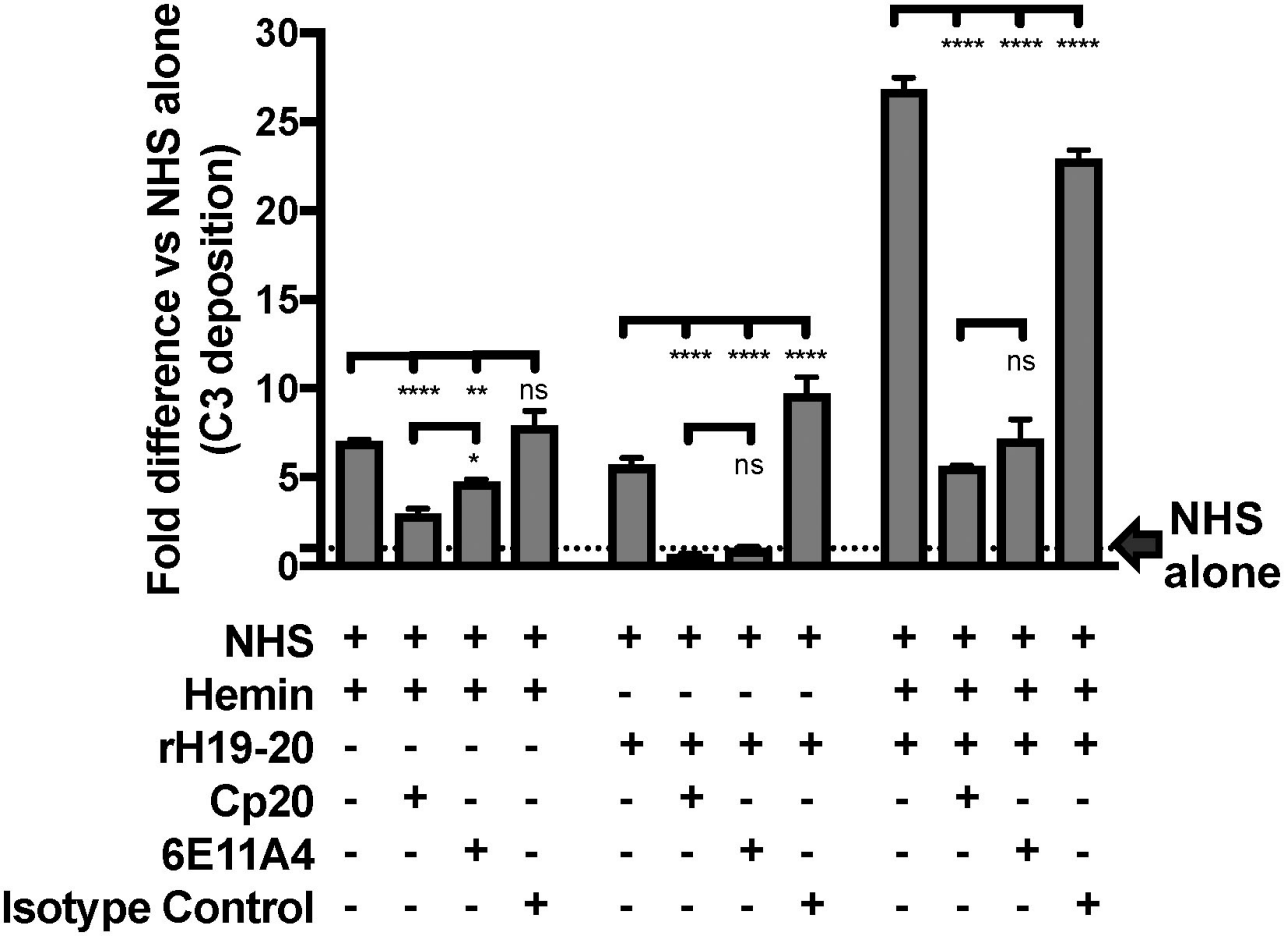
Inhibition of properdin reduces complement activation on heme-exposed endothelial cells that lack Factor H protection. HUVECs were stimulated with M199 or 100 μM hemin, washed and incubated with NHS (33% final) in the presence or absence of the following reagents at indicated final concentrations: rH19-20 (10 μM), Cp20 (50 μM), anti-properdin MoAb 6E11A4 (53 nM) or IgG1 isotype control (53 nM). C3 deposition (GMFI) was determined as described in “Materials and Methods” and representative results are graphed as the mean and SD of duplicates for each group relative to “NHS alone” (assigned value of 1; dashed line) and shown as “Fold difference.” The data were analyzed by two-way ANOVA with Tukey's multiple comparison test; ****p < 0.0001, **p < 0.01, *p < 0.05, *p* > 0.05 ns.

Finally, we aimed to determine whether properdin contributes to heme-induced complement activation on endothelial cells that lack Factor H protection (as a model for aHUS). Figure 10 (left side) shows that heme induced 7-fold C3 fragment deposition, as compared to NHS alone. The anti-properdin MoAb and Cp20 inhibited heme-induced C3 deposition by 30 and 60%, respectively, in agreement with Figure 8C. Incubation of HUVECs with rH19-20 increased C3 fragment deposition by 12-fold as compared with NHS alone (Figure 10, center) and blockade of properdin, in this case, completely eliminated rH19-20-mediated C3 fragment deposition (Figure 10, center). Blocking C3 (Cp20) also resulted in complete inhibition of C3 deposition (Figure 10, center). Heme or rH19-20-mediated increase of C3 deposits was further enhanced by over 4-fold when HUVECs were treated with both heme and rH19-20 (Figure 10, right side), and this increase was significantly attenuated by ~75% by blocking properdin or C3 cleavage (Figure 10, right side).

## Discussion

Given that properdin can be either detrimental or beneficial in disease progression, it is essential to study properdin in the context of specific pathophysiological settings. PNH and aHUS are two diseases characterized with complement AP dysregulation and current treatment with eculizumab has its limitations. Generation and characterization of the anti-properdin MoAbs, and establishment of PNH and aHUS hemolysis and endothelial cell *in vitro* models, using human samples, allowed elucidation of the critical role of human properdin, contributing to the understanding of its role in the pathogenesis of these diseases.

All the four anti-properdin MoAbs (3A3E1, 6E11A4, 1G6D2, and 6E9E6) recognized only the non-reduced form of properdin (Figure 1A), detected properdin (P_2_, P_3_, P_4_, and unfractionated properdin) in a dose-dependent manner and to a similar level (Figure 1B), and were not able to distinguish between the properdin oligomers (Figure 1B). Properdin is composed of seven thrombospondin type I repeats (TSR) [reviewed in (8)]. In an effort to map the domains of properdin that are recognized by the antibodies, we used recombinant properdin fragments in a direct ELISA and although these fragments were recognized by a polyclonal anti-properdin antibody, they were not recognized by the MoAbs (data not shown). This is likely due to the need for structural epitopes that are lost in the recombinant proteins, but remain in the non-reduced (Figure 1A; Immuno Western blot) and native (Figure 1B; direct ELISA) properdin. Anti-properdin MoAbs (3A3E1 and 6E11A4) inhibited properdin-mediated stabilization of the C3bBb convertase (Figure 1C) by inhibiting the binding of properdin to C3b (Figure 1D) in the convertase. Antibodies 1G6D2 and 6E9E6 did not inhibit hemolysis (Figure 1C) and, as expected, did not impair the binding of properdin to C3b (Figure 1D). We next carried out a competitive ELISA where 6E11A4 and 3A3E1 recognized the same or overlapping epitopes on properdin, while the non-inhibitory anti-properdin MoAbs (1G6D2 and 6E9E6) recognize distinct epitopes (Figure S1). Based on the functional assays (Figures 1C,D) and the competitive ELISA (Figure S1) data, the binding site of our inhibitory MoAbs (3A3E1 and 6E11A4) can be predicted to be in the TSR 5 or 6 domains, or to sterically inhibit these domains because previously published data has also shown (a) properdin lacking TSR5 prevents C3b and sulfatide binding (77), (b) a TSR4-5 recombinant fragment bound solid phase C3b, sulfatides, and glycosaminoglycans, but could not stabilize the C3bBb convertase (78), (c) an anti-TSR5 polyclonal antibody inhibited binding of human properdin to solid-phase C3b and blocked AP-mediated hemolysis of E_R_ (79), and (d) structural analysis of properdin reveals the importance of TSR 5 and 6 in properdin stabilization function (80, 81).

Currently approved therapies for PNH and aHUS do not inhibit upstream complement. Although significant improvement is seen in patients with C5 inhibition therapy, more than two thirds of eculizumab-treated PNH patients still experience some degree of anemia and, in some cases, remain RBC transfusion-dependent [reviewed in (26)]. Underlying mechanisms include residual intravascular hemolysis (35) and C3-mediated extravascular hemolysis (36, 38), which could be addressed by targeting upstream complement regulatory factors, such as properdin. Upstream inhibition of C3/C5 convertase formation with, for example TT30 or mini Factor H (82–84), and of C3 (85–87), properdin (19), Factor B (88), or Factor D (89), etc. is being explored by different groups for efficacy. Specifically targeting the AP in PNH and aHUS may be beneficial since it accounts for approximately 80% of the terminal pathway activity (90, 91) and may prevent both extravascular and intravascular hemolysis in PNH while preserving many of the functions of the CP and lectin pathway (LP). Since no murine PNH model is well-established [reviewed in (92)], and certain complement regulatory proteins are species specific, an *in vitro* PNH model using human samples is important to understand the effectiveness of blocking properdin and other complement components on inhibiting RBC lysis in PNH. We evaluated the role of properdin in an *in vitro* model of PNH that we previously developed (24), where an inhibitory anti-CD59 MoAb was added to the E_H_, and rH19-20 was added to amplify the sensitivity of the assay because inhibiting CD59 alone only allows a maximum of 20% lysis (24). This PNH model assay has an advantage over the traditionally used Ham's test because it eliminates the need to acidify the serum in order to obtain lysis of the erythrocytes (24). Blocking properdin protected the PNH-like RBCs from lysis and did so more effectively than blocking the other complement components when IC_90_ values are considered (Figure 2G), or in a similar manner when target/inhibitor ratios (considering properdin as a trimer) are compared (Figure 2H).

We have previously reported that Factor H protects PNH type II and III cells from complement attack, even though these cells lack GPI-anchored complement regulatory protein (CD55 and CD59) protection, and removing Factor H cell-surface protection on PNH RBCs, by adding rH19-20, lyses most of PNH type II and III cells in the presence of non-acidified serum (24). Later studies are in agreement with the important role of Factor H in protecting PNH cells (84). Using RBCs derived from PNH patients who are under eculizumab treatment, blocking properdin completely protected PNH type II and III RBCs from rH19-20-mediated lysis (Figure 3B), however eculizumab did not (Figure 3D). In agreement with our results, Gullipalli et al. (19) show that inhibiting properdin protects acidified serum-mediated human PNH RBC lysis, while Sica et al. has shown partial inhibition by eculizumab *in vitro* (23). In addition, blocking properdin, which inhibits early AP activity, prevented C3 fragment deposition on PNH RBCs from PNH patients with or without eculizumab treatment, while eculizumab did not, as expected (Figure 3E). In line with this, others have shown that anti-properdin MoAbs can prevent extravascular hemolysis of complement-susceptible RBCs in a murine model by inhibiting C3 fragment opsonization (19). Moreover, anti-properdin MoAbs had the lowest IC_90_ values (Figure 4G) and similar target/inhibitor ratio values (Figure 4H) as compared with the respective values obtained by blocking other complement components for protecting the PNH patient RBCs from hemolysis. Additionally, the IC_90_ values of these complement inhibitors on PNH RBCs were very similar to what was obtained using the “PNH-like” RBCs (Figure 2), supporting the use of the *in vitro* PNH model to evaluate other potential complement inhibitors. Eculizumab and tick-derived OmCI/coversin were not able to abolish PNH RBC lysis in our assay (Figures 3D, 4E–G). This is expected and is intrinsic to the nature of these C5 inhibitors, as others have shown that the high density of C3b on eculizumab-treated PNH RBCs compete with C5 inhibitors (such as eculizumab and OmCI) for the binding to C5, allowing C5 cleavage and PNH RBC lysis, while the combination of two C5 inhibitors overcomes the incomplete protection (35). 40% fresh NHS and MgEGTA in our assay would facilitate the AP activation and generate a high density of C3b on PNH RBCs, even in the presence of C5 inhibitors. Observations of residual hemolysis (Figure 4E) indicate that this assay could be used for evaluating other complement inhibitors on their ability to overcome residual hemolysis. Surprisingly, the residual hemolysis achieved with eculizumab also occurred when evaluating *in vitro* lysis of PNH RBCs from patients not on eculizumab treatment (Figure 5). In these hemolysis assays, rH19-20 is added to the NHS to enhance AP activity against PNH type II and III cells (instead of acidifying the serum) (24). Eculizumab had marginal effects on protecting these RBCs from AP-mediated hemolysis while anti-properdin MoAbs inhibited the lysis over 50% (Figure 5). This data indicates that strong AP activation can be suppressed by inhibiting properdin, while it overwhelms the ability of eculizumab to protect, similar to what was observed with PNH patients under eculizumab treatment. Furthermore, the combination of a saturating dose of eculizumab (where residual hemolysis was still observed) and a sub-maximal dose of an anti-properdin MoAb (where 50% of the RBCs from patients under eculizumab treatment were protected from hemolysis) significantly inhibited the lysis that eculizumab alone cannot eliminate (Figure 6). This indicates that co-administration of anti-properdin MoAbs and eculizumab to PNH patients with suboptimal responses to eculizumab may be practical in a switch treatment approach, as researchers have suggested for an anti-Factor D inhibitor (93). Inhibiting the AP alone may not be sufficient in certain scenarios, as has been shown in *in vitro* models testing the effect of absence of Factor B in the presence of anti-RBC alloantibodies, which induce strong classical pathway activation (35); alloantibodies have been found in a few PNH patients (94). However, inhibiting properdin alone (as monotherapy) may be sufficient in most PNH scenarios by inhibiting the causal AP-mediated damage, while still preserving the other two pathways (CP and LP), and may be a potential alternative treatment to eculizumab. The possibility of using inhibition proximal to C5 as a monotherapy is supported by *in vitro* data using TT30 (83) and Cp40 (86). Altogether, the data support the need for clinical studies to validate the *in vitro* data.

A high proportion of aHUS cases are caused by mutations in the C-terminus of Factor H that impair the ability of Factor H to bind to cells and protect them. Unlike membranoproliferative glomerulonephritis that is caused by total Factor H deficiency, fluid phase complement regulation remains intact in aHUS [reviewed in (95)]. Ueda et al. showed that blocking properdin ameliorated the pathology in an aHUS mouse model that carries a Factor H point mutation (W1206R) (50). In order to address the importance of human properdin and how properdin contributes to the pathology of human aHUS, we first evaluated the role of properdin in an *in vitro* aHUS RBC model that was previously developed in our lab using rH19-20 (24, 56). Blocking properdin protected the RBCs that lack Factor H protection from lysis at least as effectively as blocking the other complement components when IC_90_ values are considered (Figure 7G), or as effective as blocking C5 and less effective than blocking Factor B when target/inhibitor ratios (considering properdin as a trimer) are compared (Figure 7H). The data indicate that properdin is required for AP-mediated lysis of these RBCs and that blocking AP proteins were effective at inhibiting lysis in this system.

We next sought to determine the contribution of properdin to heme-induced complement activation. Excessive and chronic hemolysis occurs under various pathological scenarios and the released heme activates AP on cell surfaces by down-regulating expression of membrane-bound complement regulatory proteins (CD55 and CD46) and upregulating P-selectin (66) on endothelial cells, contributing to overall pathology [reviewed in (96, 97)]. Figure 8C shows that blocking properdin significantly inhibited complement activation on endothelial cells in the presence of hemolysis-derived heme, indicating that properdin may play a role in heme-induced endothelial cell pathology. Interestingly, completely inhibiting C3 using Cp20 (Figure 8) or EDTA (Figure 8B), did not fully inhibit C3 fragment deposition as compared with NHS alone, indicating that a portion of C3 products was captured by endothelial cells independent of complement activation. This finding agrees with the model proposed by (66), where heme induces upregulation of P-selectin (98), which has been shown by others to bind C3b and C3(H_2_O), serving as an initiating point for AP activation on endothelial cells and platelets (99, 100).

Others have used aHUS patient sera with heme-exposed endothelial cells to define the essential roles that aHUS mutations and heme play on endothelial cell pathology (66). In order to define the role of properdin in aHUS-mediated complement activation on endothelial cells, we established a modified assay that does not require patient serum, but relies on inhibiting Factor H-mediated protection of cell surfaces by using a competitive inhibitor of Factor H (rH19-20). First, to confirm the importance of Factor H protection on endothelial cells (74, 76), we standardized our assay without heme, in the presence of rH19-20 or rH19-20 mutants. Figures 9A–C show that rH19-20 alone increased C3 deposition as compared with NHS alone group by 7 to 11-fold, despite the presence of other complement regulatory proteins on endothelial cells. In addition, mutations that have been previously shown to be highly (R1215Q and W1183L), intermediately (L1189R), or minimally impaired (T1184R) in their ability to compete with full length Factor H on various cell surfaces (18, 57, 76) showed similar variation in C3 fragment deposition on the endothelial cells (Figures 9A–C) to that previously described. Because heme would be present in aHUS conditions due to excessive hemolysis, we next examined how these aHUS-related mutations affect complement activation on endothelial cells in the presence of heme. Figures 9D–F show that addition of rH19-20 significantly enhanced heme-induced C3 fragment deposition by 2-3-fold and the mutations showed similar impairment variations in the presence of heme (Figures 9D–F) as that in the absence of heme (Figures 9A–C). Thus, impairment of Factor H protection enhances complement dysregulation and causes C3 fragment deposition on endothelial cells. Using this aHUS *in vitro* model, we next evaluated the contribution of properdin to the C3b deposition. C3b deposition is reduced on heme+NHS-exposed endothelial cells by ~40% when properdin is inhibited and by ~60% when C3 is inhibited (Figure 10, left side; Figure 8C). The reduction of C3b deposition by blocking properdin or C3 becomes highly effective and equivalent to each other when endothelial cells are exposed to NHS+rH19-20 alone (100% inhibition; Figure 10, center) or with heme+NHS+rH19-20 (~75%; Figure 10, right side), indicating that all complement activity on rH19-20-exposed endothelial cells was due to the AP, as we have shown on RBCs or platelets (18, 56). Thus, under the “aHUS-like” condition (Figure 10, right side), properdin is playing a central role in promoting complement activation on endothelial cells. Properdin is essential for proper stabilization of the AP C3 convertases and thus, aHUS patients with gain-of-function mutations in Factor B (101) or C3 (102), which already have highly stable C3-convertases, may not benefit from blocking properdin and may require intervention at later stages of the cascade (i.e. C5 inhibition). However, inhibition of efficient convertase function by blocking properdin may benefit patients with loss-of-function aHUS-associated mutations by reducing C5b-9 deposits on microvascular endothelial cells, which have been correlated with aHUS relapse (103), in addition to preventing formation of pro-inflammatory mediators C3a and C5a. Identifying a non-antibody-mediated alternative for inhibiting properdin function would be ideal to avoid theoretical complications associated with potential antibody binding to properdin that may already be bound to convertases on RBCs (such as serving as opsonins on the RBCs or sterically inhibiting Factor H binding to the RBCs), as has been shown when using anti-C3 for C3 inhibition (104).

In conclusion, our study characterized inhibitory and non-inhibitory anti-properdin MoAbs, which allowed identification of a critical role of human properdin in PNH and aHUS pathogenesis in *in vitro* models developed in our laboratory. Inhibition of properdin prevented hemolysis and complement activation on human endothelium, such as that observed in hemolytic and thrombotic disorders with AP dysregulation. Inhibition of properdin was at least as, or more, efficient as other complement inhibitors that were tested. Thus, clinical studies aimed at evaluating properdin inhibition as an alternative treatment strategy for these diseases are warranted. Our *in vitro* RBC-based assays described herein have the advantage of being able to test the complement inhibitors using human samples. The *in vitro* endothelial cell aHUS assay, by not requiring patient samples, may serve as a convenient way of screening the efficacy of other complement inhibitors in this disease model.

## Data Availability Statement

The raw data supporting the conclusions of this article will be made available by the authors, without undue reservation, to any qualified researcher.

## Ethics Statement

The studies involving human participants were reviewed and approved by Institutional Review Board University of Toledo College of Medicine and Institutional Review Board John Hopkins University. The patients/participants provided their written informed consent to participate in this study.

## Author Contributions

Experiments were designed by JC, NG, HE, SSM, VF, and conducted by JC, NG, HE, and SSM. Key reagents were generated and provided by CC, JT, SAM, and RB. The manuscript was written by JC, NG, HE, and VF. All authors critically reviewed the manuscript and made key contributions to the analysis and interpretations of the results.

## Conflict of Interest

JT receives royalties from Alexion Pharmaceuticals, Inc. JT was also a consultant for and receives royalties from AdMIRx, Inc., a company developing complement inhibitors. RB was on the scientific advisor board for both Alexion and Achillion pharmaceuticals. RB receives grant funding (PNH registry) from Alexion Pharmaceuticals. The remaining authors declare that the research was conducted in the absence of any commercial or financial relationships that could be construed as a potential conflict of interest.

## Abbreviations

AHUS: atypical hemolytic uremic syndrome
AP: alternative pathway
EDTA: ethylene diamine tetraacetic acid
EGTA: ethylene glycol tetraacetic acid
ELISA: enzyme linked immunosorbent assay
E_H_, E_R_, E_S_: human, rabbit, sheep erythrocytes respectively
E_S_C3b: sheep erythrocytes coated with C3b
GMFI: geometric mean fluorescence intensity
GPI: glycosylphosphatidylinositol
GVB: gelatin veronal buffer
HUVECs: human umbilical vein endothelial cells
IC_50_: half maximal inhibitory concentration
IC_90_: 90% maximal inhibitory concentration
MAC: membrane attack complex
MoAb: monoclonal antibody
NHS: normal human serum
P_2_: dimeric form of properdin
P_3_: trimeric form of properdin
P_4_: tetrameric form of properdin
PBS: phosphate buffered saline
PNH: paroxysmal nocturnal hemoglobinuria
RBC: red blood cell
rH19-20: recombinant domains 19-20 of Factor H
TSR: thrombospondin type I repeat.
