# Supplementary material for: Properdin Is a Key Player in Lysis of Red Blood Cells and Complement Activation on Endothelial Cells in Hemolytic Anemias Caused by Complement Dysregulation
Source: Front Immunol. 2020 Jul 22;11:1460. doi: 10.3389/fimmu.2020.01460 (PMC7387411; doi:10.3389/fimmu.2020.01460)
Supplement: Supplementary file 1 [file Data_Sheet_1.docx]

Supplementary Material


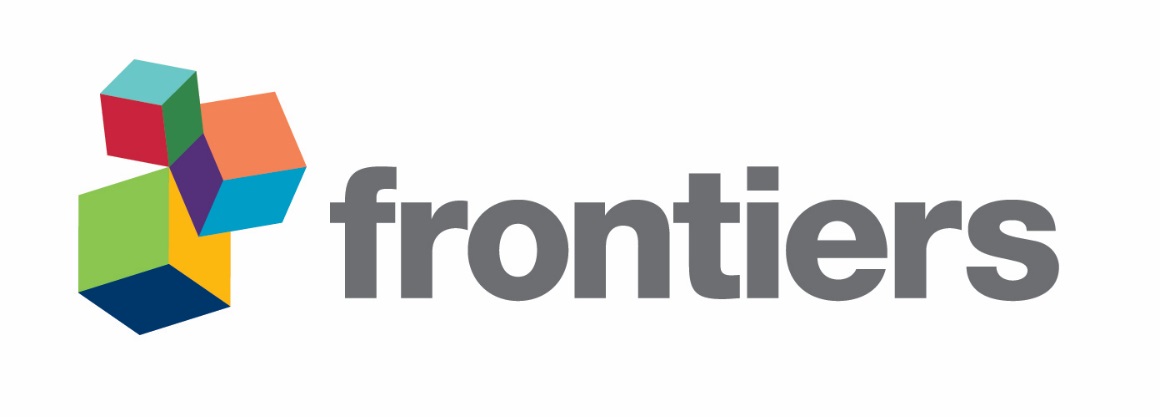


## Supplementary Methods

**Supplementary Method 1. Competitive ELISA for determining whether the anti-properdin MoAbs recognize similar or distinct epitopes on properdin**

A competitive ELISA was performed as described in manuscript “Materials and Methods” section “Direct ELISA”, with the following changes: plates were coated with 160 ng/ml pure unfractionated properdin, MoAbs 6E9E6, 6E11A4, 3A3E1, and 1G6D2 were biotinylated using a kit following manufacturer’s instructions (Thermo Scientific) and used at 40 ng/ml in a mix with increasing concentrations of the unlabeled MoAbs (0-400 ng/ml) in PBS/1% BSA/0.05% Tween. The mix was immediately added to the properdin-coated wells (100 µl/well) and horseradish peroxidase-streptavidin (Biolegend) at 1/1,000 dilution (100 µl/well) was used for detection of the remaining bound biotinylated antibody.

## Supplementary Figures

**
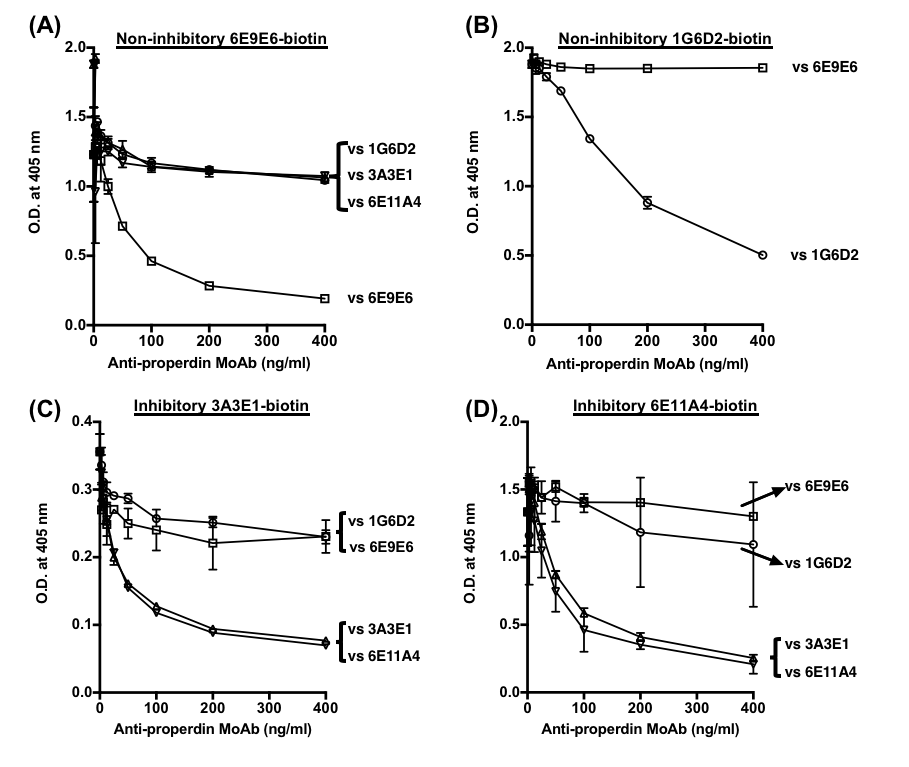
**

**Figure S1** Non-inhibitory anti-properdin MoAbs 1G6D2 and 6E9E6 recognize distinct epitopes on properdin. **(A)** Biotinylated 6E9E6 or **(B)** biotinylated 1G6D2 or **(C)** biotinylated 3A3E1 or **(D)** biotinylated 6E11A4 at 40 ng/ml were mixed with serial dilutions of one of the unlabeled MoAbs (0-400 ng/ml) to perform the competitive ELISA (as described in Supplementary Method 1). Representative results are graphed as mean and SD of duplicate observations.

**
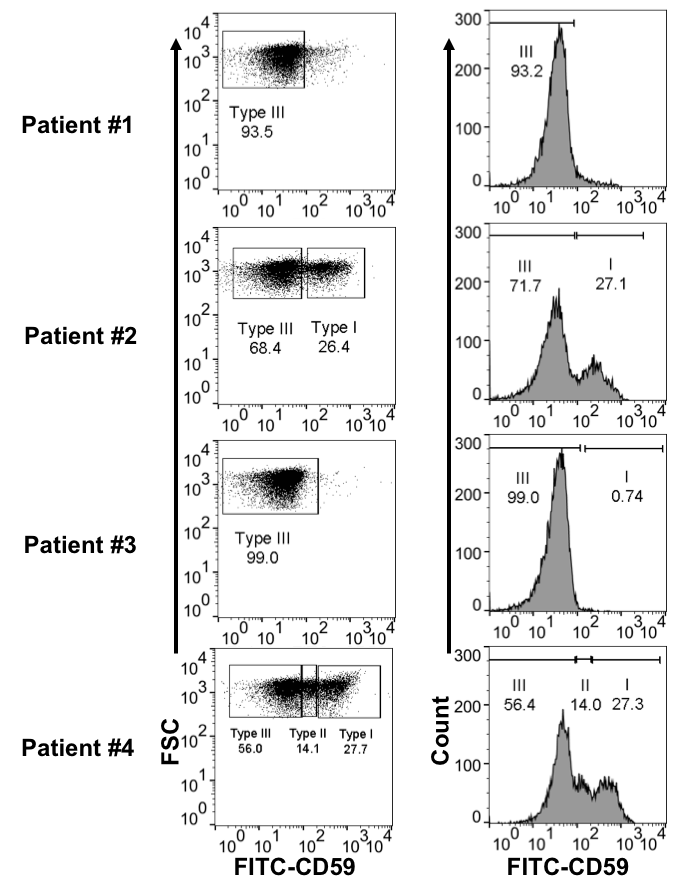
**

**Figure S2** CD59 profile of RBCs derived from four PNH patients (determined as described in “Materials and Methods” section “CD59 and C3 Fragment Profile Analysis of PNH RBCs After Exposure to NHS and rH19-20 in the Presence or Absence of Anti-Properdin MoAbs or Eculizumab”). The markers I, II and III indicate the populations of normal RBCs, type II and type III PNH RBCs, respectively.
